# Recognition Rate Advancement and Data Error Improvement of Pathology Cutting with H-DenseUNet for Hepatocellular Carcinoma Image

**DOI:** 10.3390/diagnostics11091599

**Published:** 2021-09-02

**Authors:** Wen-Fan Chen, Hsin-You Ou, Cheng-Tang Pan, Chien-Chang Liao, Wen Huang, Han-Yu Lin, Yu-Fan Cheng, Chia-Po Wei

**Affiliations:** 1 Institute of Medical Science and Technology, National Sun Yat-sen University, Kaohsiung 80424, Taiwan; sallychen@imst.nsysu.edu.tw; 2 Liver Transplantation Program and Departments of Diagnostic Radiology, Surgery Kaohsiung Chang Gung Memorial Hospital, Chang Gung University College of Medicine, Kaohsiung 833401, Taiwan; ouhsinyou@gmail.com (H.-Y.O.); liao1009@gmail.com (C.-C.L.); 3 Department of Mechanical and Electro-Mechanical Engineering, National Sun Yat-sen University, Kaohsiung 80424, Taiwan; pan@mem.nsysu.edu.tw (C.-T.P.); hwkakaku@mem.nsysu.edu.tw (W.H.); hanyu@mem.nsysu.edu.tw (H.-Y.L.); 4 Department of Electrical Engineering, National Sun Yat-sen University, Kaohsiung 80424, Taiwan

**Keywords:** data comparison, deep learning, H-DenseUNet, lesion segmentation, liver segmentation, medical statistics, pathological

## Abstract

Due to the fact that previous studies have rarely investigated the recognition rate discrepancy and pathology data error when applied to different databases, the purpose of this study is to investigate the improvement of recognition rate via deep learning-based liver lesion segmentation with the incorporation of hospital data. The recognition model used in this study is H-DenseUNet, which is applied to the segmentation of the liver and lesions, and a mixture of 2D/3D Hybrid-DenseUNet is used to reduce the recognition time and system memory requirements. Differences in recognition results were determined by comparing the training files of the standard LiTS competition data set with the training set after mixing in an additional 30 patients. The average error value of 9.6% was obtained by comparing the data discrepancy between the actual pathology data and the pathology data after the analysis of the identified images imported from Kaohsiung Chang Gung Memorial Hospital. The average error rate of the recognition output after mixing the LiTS database with hospital data for training was 1%. In the recognition part, the Dice coefficient was 0.52 after training 50 epochs using the standard LiTS database, while the Dice coefficient was increased to 0.61 after adding 30 hospital data to the training. After importing 3D Slice and ITK-Snap software, a 3D image of the lesion and liver segmentation can be developed. It is hoped that this method could be used to stimulate more research in addition to the general public standard database in the future, as well as to study the applicability of hospital data and improve the generality of the database.

## 1. Introduction

According to statistics, chronic liver disease and liver cancer are among the leading causes of death each year. Hepatocellular carcinoma (HCC) is mainly caused by primary malignant tumors of the liver and is commonly seen in patients with chronic liver disease or cirrhosis following hepatitis B and C infection and has fewer early symptoms. Traditionally, radiologists often need to collect a lot of patient information and review each magnetic resonance imaging (MRI) report separately, which not only generates a huge workload but also may delay the optimal treatment time for liver cancer patients. In recent years, artificial intelligence (AI) has been able to predict liver lesions as well as avoid its deterioration. Further, the use of AI can not only reduce the significant expense required for tomography but also reduce the rate of misdiagnosis of patients. In the medical field, Klinder et al. [1] used a comprehensive means of defining characteristic items after performing automatic segmentation applied to vertebra computed tomography (CT) images and proposed the concept of automatic segmentation. In the machine learning field, Erickson et al. [2] provided a detailed introduction to the various concepts and processes of machine learning. Giger et al. [3] illustrated the future development of machine learning for medical imaging applications. Robinson et al. [4] used the application of machine learning for breast cancer risk assessment. Wernick et al. [5] proposed various ways of using machine learning in medical imaging and demonstrated them through different examples. Pratondo et al. [6] utilized machine learning integrated with region-based active contour models to improve its accuracy and speed. Although the later proposed machine learning techniques [2,3,4,5,6] have achieved a breakthrough in recognition rate, they are less adaptable and more time-consuming to recognize objects because of the need to build features manually first.

In recent years, due to the rise of deep learning technology [7,8], the use of this method to identify various image features and applications in medical imaging has been increasing [9,10,11]. In terms of the recognition architecture, the rectified linear activation function or rectified linear unit (ReLU) [12] proposed in 2011 solved the problem of gradient disappearance and laid the foundation for future neural networks. Further, the improvement of AI deep learning has attracted a lot of attention because the error rate of AlexNet [13] in ImageNet [14,15] has increased significantly. The deep residual network (ResNet) [16], proposed in 2015, surpassed human performance in visual classification for the first time. ResNet deals with the problem of gradient disappearance and gradient over-expansion in the deep CNN [17,18] structure, making the residual block a fundamental structure in the CNN structure. In 2015, the UNet [19] proposed by Ronneberger et al. led the trend for more deep learning. The partitioned network is similar to fully convolution networks (FCNs) [20], and compared to FCNs, UNet is characterized as fully symmetric. The decoder of FCNs is simpler than that of UNet, using only the deconvolution action, and does not use the convolution structure. Further, UNet uses a concatenation operation, while FCN uses a summation operation.

In terms of database creation, Poon et al. [21,22,23] proposed livewire and similar automatic edge curve fitting functions and their algorithms to reduce the manual time and segmentation time in building mask databases, which have significantly improved the creation rate of the database. In 2016, Cho et al. [24] presented a study on the impact of the number of hospital data training sessions on the recognition rate, which provides a standard for the number of patients required to build a database. The Fusion-Net proposed in 2019 by Kan et al. [25] is particularly suitable for a general fusion unit of hand-drawn segmentation. In 2021, Rutherford et al. [26] conducted a study on auto-mask failure due to poor quality raw data and investigated the identification errors caused by the quality and condition of medical images.

Automatic segmentation of the liver and tumor from images without contrast is a very difficult task because the tumor boundaries in the images are not as clear as in normal images. Many deep learning recognition systems use normal 2D digital imaging and communications in medicine (DICOM) files [27,28] and/or JPEG files [29]; however, the performance is not as good as the 3D recognition solutions [30,31]. In the clinical diagnosis, radiologists usually view tumor images slice by slice, combining information from the previous slice and the next slice to evaluate the tumor, which is one of the main reasons why the 2D solution does not perform as accurately as the 3D solution. However, the drawback of 3D networks is that they require a lot of random access memory (RAM) and training time, so by using the H-DenseUNet [32], the RAM and training time requirement would be significantly reduced. In addition, another advantage of using H-DenseUNet is that it obtained excellent recognition results in the LiTS challenge, and the ability to reduce training time and output liver segmentation at the same time met our needs. One of the features of the hybrid 2D/3D training module is that the 3D layer information is recorded into the training module. During training, if a tumor is detected in the upper layer, the same location in the next layer will localize the tumor more efficiently. Further, the non-contrast media and the different contrast stage of contrast media should be mixed together to ensure optimal accuracy. This will lead to differences in the color of the lesions, which in turn will lead to a decrease in recognition rate during recognition training.

Recent studies have focused on the comparison of recognition rates using public datasets, and relatively few studies have compared the results of recognition output to medical pathology data. Therefore, this study will be conducted to understand the improvement effect achieved by mixing the data and spending a small amount of training time on the hospital database and also to understand the status of the discrepancy between the recognition results and the pathological data. By using the image and pathology data from the medical center, we can compare the error between the automatic recognition and the pathology data after recognition and the difference in recognition difficulty between the LiTS [33] database and the medical center database. By importing the database of Kaohsiung Chang Gung Memorial Hospital into the training, the error rate of the system identification can be reduced. Since the densely vascularized area of the liver is often the area that is more prone to misclassification during identification, the problem can be improved by importing more hospital data, and the lesions that have gone through RFA/post TAE can be identified with higher capture performance. Further, in the hospital, the 3D displays of the DICOM viewer software provided by the device company are not perfect. By using the ITK-SNAP [34] in combination with software such as 3D slice [35,36,37,38] for the tumor segmentation, physicians would have a clear 3D tumor display [39] with adjustable liver transparency. The use of a 3D slice will be able to show, in addition to the segmentation of the lesion, the distribution status of blood vessels within the liver as well as the volume and proportion of the tumor and liver. Further, the mean density of the tumor and liver will be calculated by the Hounsfield unit [40], while the longest side of the tumor [18] will also be calculated to give the radiologist as much information as possible when evaluating the tumor.

## 2. Proposed Method

### 2.1. Selection and Input of Data

**Figure 1:**
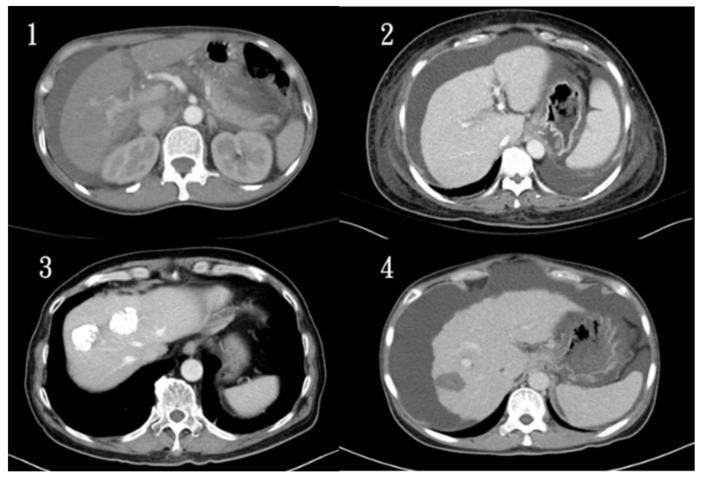
Common problems of medical imaging in current medical centers (1. normal images, 2. contrast-delay phase, 3. post-TAE, and 4. RFA).

Currently, medical images used in medical centers often have several problems (with and without contrast media, post-transcatheter arterial embolization (TAE), and radiofrequency ablation therapy (RFA), as shown in Figure 1), so it is important to select the image files for input training and test recognition results. A total of 500 patient data provided by Kaohsiung Chang Gung Memorial Hospital were reduced to 100 patients. In this section, less complex cases from the images were selected, the files with more perihepatic disturbances and very small or difficult to identify lesions were removed, and the files with incomplete pathological data were also deducted, for a total of 70 patients. The image files with various conditions were mixed. The original medical images are in the DICOM standard sheet format, which contains the original medically relevant data (length, thickness per layer, and housefield unit). After conversion using the MRI converter (Lewis Center for Neuroimaging, University of Oregon v.2.1.0) [41] to a 4D neuroimaging informatics technology initiative (NIfTI) file, the original data were still retained. Then, the 70 patients were divided into a testing group of 40 patients and a training group of 30 patients, and the original LiTS database was mixed, with 30 groups for training. For the identified targets tumor, post-TAE and RFA, the treated and untreated parts will be identified.

### 2.2. Overview of Whole Medical Center Identification Error Comparison Statistics System

**Figure 2:**
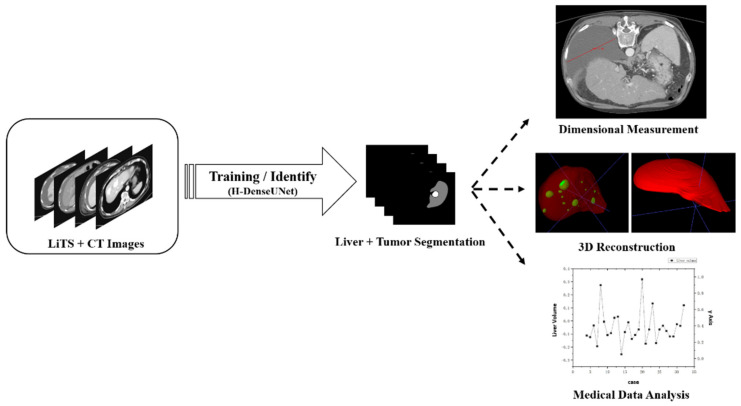
Process of the hospital pathology data error comparison statistical system.

The process of the hospital pathology data error comparison statistical system is shown in Figure 2. The system is divided into two stages: the first stage uses H-DenseUNet for training and recognition, and the result will be output as a NIfTI file for segmentation. In the second stage, the original CT images and the segmented lesion segmentation files will be combined and imported into ITK-SNAP (v.3.8.0) [34], LIFEx (v.7.0.0) [42,43] and 3D-slicer (v.4.11.20210226) for 3D modeling and data analysis. Finally, the data will be compared with the pathology data of Kaohsiung Chang Gung Memorial Hospital to obtain the average error rate of the data.

### 2.3. Stage 1—H-DenseUNet

**Figure 3:**
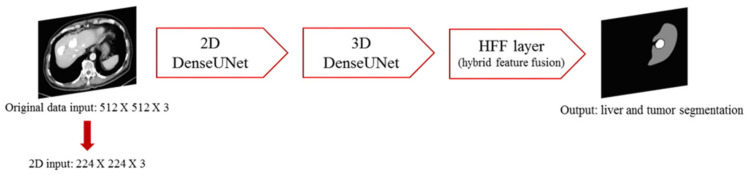
Identification process of H-DenseUnet.

In the first stage, the H-DenseUnet model proposed by Li et al. [32] was used for training, which combines the faster capability of 2D with the high accuracy of 3D. A total of 101 patients from the LiTS database and 30 patients from the Chang Gung Memorial Hospital database were used for the training. The original CT image data is 512 × 512 resolution, and the resolution was automatically adjusted to 224 × 224 resolution for convolutional input during the input process. Each 3D input image file was sliced into adjacent slices and imported into ResNet for initial region of interest (ROI) extraction. The segmented liver and tumor slices were obtained and imported into 2D DenseUnet for 2D Intra-slice feature extraction. The image files continued to be imported into 3D DenseUnet for extracting inter-slice features, and the hybrid feature fusion (HFF) layer was fused and the intra-slice and inter-sliced features optimized to precisely identify the liver and the lesion locations. The architecture is shown in Figure 3.

### 2.4. Stage 2—Recognition Result Data Acquisition, Analysis Method and 3D Image User-Friendly Display Method

**Figure 4:**
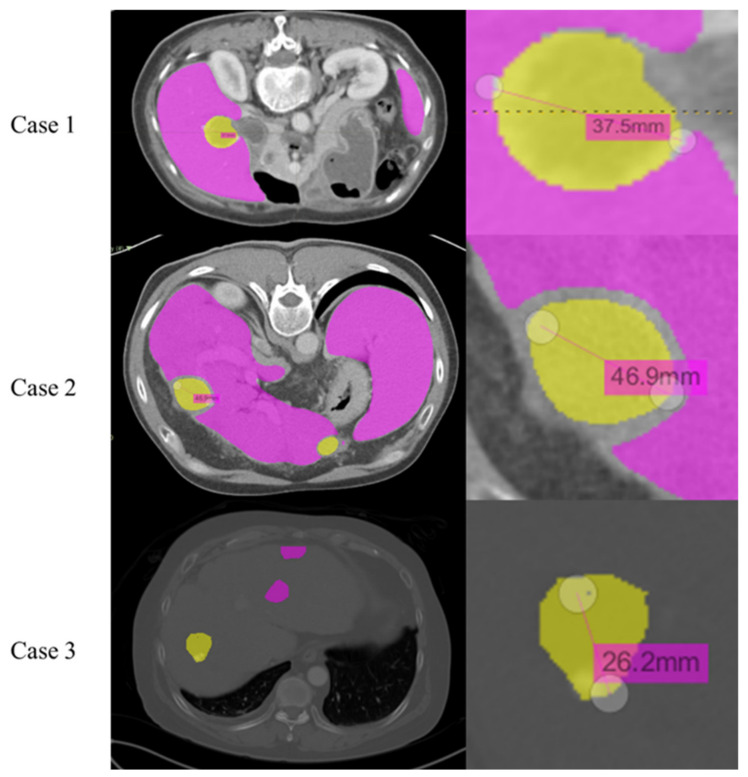
Interface image for measuring liver and lesion length in the LIFEx program.

The output data were imported into the LIFEx program to measure the length of the longest side of the liver and the length of the lesion, as shown in Figure 4. After superimposing the output segmentation data to the original CT medical image, the measurement was performed using the values embedded in the original medical image DICOM.

The lesion and liver volumes in the database were measured using the ITK-Snap software. Table 1 shows how the lesion and liver volume are displayed in the software interface. The voxel count represents the total number of volume pixels contained in the label, volume (mm^3^) represents the calculated value of the volume of the label, and intensity mean ± SD represents mean image intensity inside the structure.

**Figure 5:**
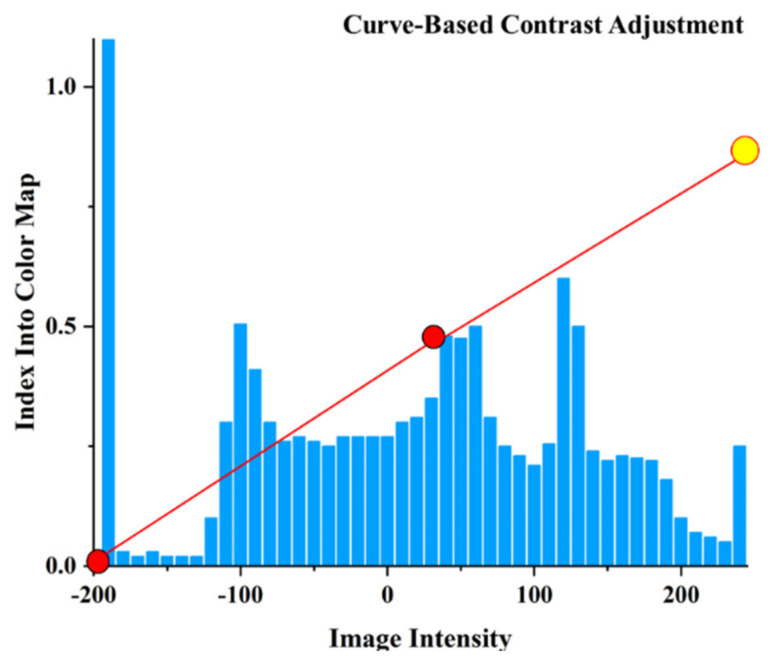
Image intensities mean, range, and distribution histogram of the lesion and liver area shown in the program (the red line and markers are used to adjust the view screen, which can be used to adjust the display intensity of each image intensity).

Figure 5 shows the interface and the presentation of the average and upward and downward fluctuations of the image intensity of the lesion area and the liver area, as well as the histogram of the distribution of the image intensity values in the program. Because of its similarity to Hounsfield units, it has a complementary function in medical assessment. Further, the intensity and range of the image intensity can be adjusted and highlighted by pulling the red and yellow dots.

**Figure 6:**
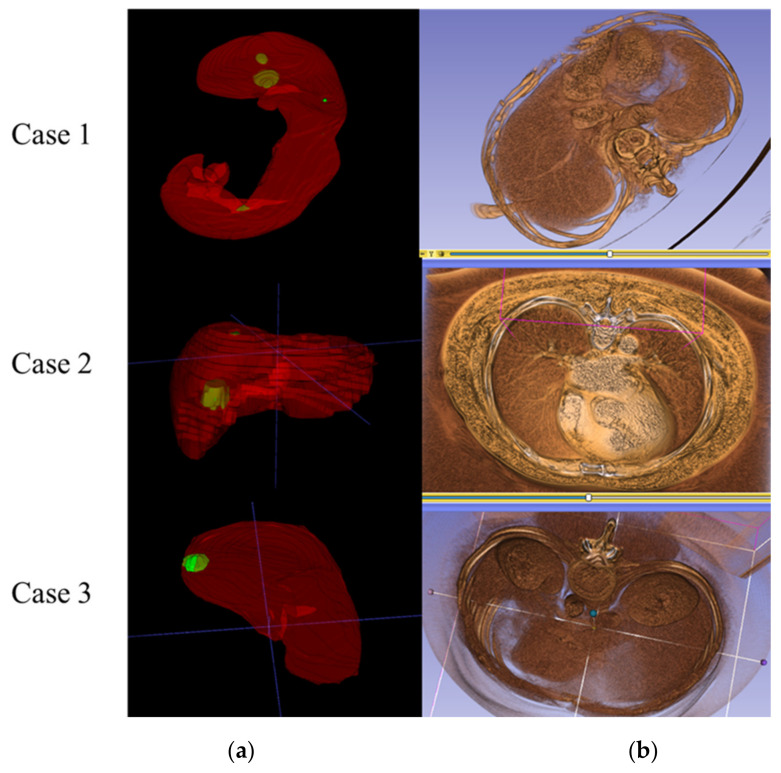
Three-dimensional segmentation of (**a**) ITK-Snap and (**b**) 3D slicer for medical image display.

The presentation of the output 3D medical images can be performed by ITK-Snap and 3D slicer software. The ITK-Snap image is a translucent display of the combined liver and lesion. For the 3D slicer image, it can not only display the liver and lesion segmentation with the original CT image in a semi-transparent manner but also show the distribution of arteries and blood vessels in the liver simultaneously. Further, the 3D slicer image can blend the blood vessels and textures contained in the liver from the original medical image with the patient’s skeletal structure for further interpretation. The images presented by the two systems are shown in Figure 6.

## 3. Liver CT Dataset

The mask and lesion mediations were annotated and plotted by Kaohsiung Chang Gung Memorial Hospital (KCGMH) radiologists and manually plotted on a sheet-by-sheet basis using LIFEx software. The medical imaging interaction toolkit (MITK) [44] was used to draw the pixel-edge fit with similar automatic pixels. The image files of 70 patients with various characteristics were obtained by random selection of 400 patients’ data obtained from the hospital and combined with 101 data obtained from LiTS for use. To import hospital dicom data (usually 2D dicom files) for training or recognition, software was required to convert the dicom format to 4D nifti format [45,46]. In this study, MRI Convert was used to convert the file, and the nifti files need to include the z-axis orientation and voxel spacing data.

## 4. Results and Discussion

### 4.1. Training Method and Environment

Each training image set contains raw CT, a liver mask, and a tumor mask. This part of the training was divided into 2D and 3D parts. A total of 55 epochs were used on training 2D Resnet/3D DenseUNet, and a total of 40 h were spent on training. Stage-1 of training and testing was implemented on the environment: Intel^®^ Core™ i7-9700KF CPU, DDR4 128GB memory, RTX 2080 Ti GPU, and Windows 10. The software was Keras 2.3.0 and Python 3.7.7. Further, Stage-1 output includes liver and lesion parts together in the same segmentation file, so the two stored values are different, and liver and lesion abnormalities can be counted separately.

### 4.2. Evaluation Metrics

The performance of the proposed Stage-1 after training with a mixture of hospital data was evaluated by Taha et al. [47], and the correct answers were compared using their proposed recognition rate calculation program. The formula is also based on the evaluation formula and features proposed by Taha et al. [47]. In this study, Intersection over Union (IoU) (as known as Jaccard coefficient), Accuracy (ACC), Area Under the ROC Curve (AUC), Dice coefficient, and Average Hausdorff Distance (AVGDIST) [48] were used as metrics. These metrics can be composed of the following four parameters: TP (true positive), TN (true negative), FP (false positive), and FN (false negative) by means of the values embedded in the segmentation. The values of segmentation are 0, 1, and 2, where 0 represents the site of no liver and lesion, 1 represents the site of liver, and 2 represents the site of lesion. The equation for accuracy is as follows:

(1) $$\mathrm{Accuracy}=\frac{\mathrm{TP}+\mathrm{TN}}{\mathrm{TN}+\mathrm{FP}+\mathrm{TP}+\mathrm{FN}}\;$$

Further, the ACC is mainly used to compare the value of each point and calculate the correct ratio. However, due to the nature of medical image segmentation, the black areas occupy a larger area, so the average ACC value is higher and thus the variability is reduced. For IoU, the equation is as follows:

(2) $$\mathrm{IoU}=\;\frac{\mathrm{TP}}{\mathrm{FP}+\mathrm{TP}+\mathrm{FN}},$$

Compared with ACC, IoU does not have a TN part, thus eliminating the problem of high values in ACC, which is a more commonly used evaluation method. Further, the AUC is obtained from the ROC curve, and the ROC value is obtained by the true positive rate TP/(TP + FN) against the false positive rate FP/(FP + TN) at various threshold settings. The higher the AUC, the better the performance of the model at distinguishing between the positive and negative classes. For the Dice coefficient value, which is two multiplied by the area of overlap divided by the total number of pixels in both images as follows:

(3) $$\mathrm{DSC}=\frac{2\mathrm{TP}}{\mathrm{FP}+2\mathrm{TP}+\mathrm{FN}},$$

The Hausdorff distance is a measure of the distance between two subsets of a metric space from each other, which is commonly used in medical image segmentation. The average Hausdorff distance value is shown below:

(4) $$\mathrm{Average}\;\mathrm{Hausdorff}\;\mathrm{distance}=(\frac{\mathrm{GtoS}}{G}+\frac{\mathrm{StoG}}{S})/2\;$$

where GtoS is the directed average Hausdorff distance from image ground truth to segmentation, and Hausdorff distance is the image ground truth that has to travel from a point in image ground truth to its closest point in the segmentation. Taking the average of these is GtoS. StoG is the directed average Hausdorff distance from segmentation to the ground truth image. Taking the average of these is StoG. Both units are calculated using the number of pixels. G is the number of voxels in the ground truth, and S is the number of voxels in the segmentation.

### 4.3. Recognition Rate of Liver and Lesions

**Figure 7:**
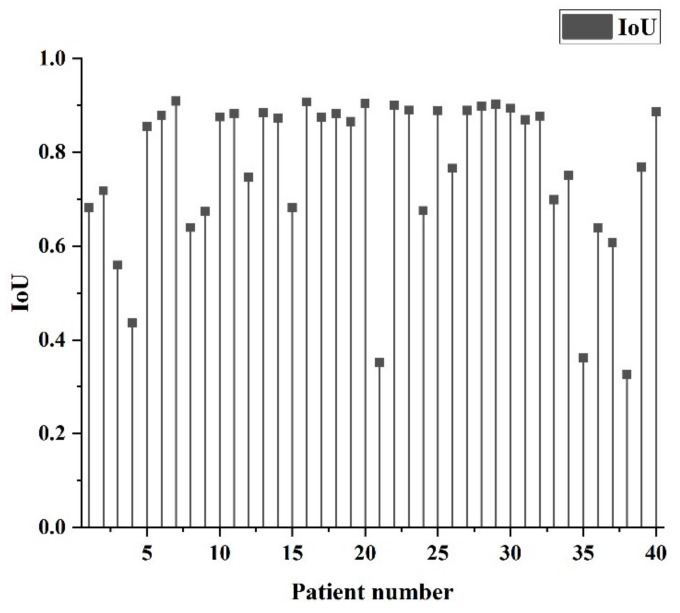
Distribution of IoU by patients after training with the default LiTS database.

Table 2 shows the recognition rate of H-DenseUNet applied to identify liver and lesions after LiTS training of hospital data, and the IoU distribution of individual patients is shown in Figure 7. Since the IoU index is the ratio of the area of interaction to the area of union, it is more comparable in images with more black areas. The AUC value obtained for the liver site is 0.967, which indicates that the predictive value of this model is excellent. However, the value obtained for the lesion site was lower than that for the liver site, which is 0.781. This value is acceptable considering that the recognition difficulty in the image is higher than the general database. Further, the numerical fallout of AVGDIST shows that the deviation of the lesion identification location is still quite high. In IoU, AUC, and AVGDIST, it can be understood that since the lesion area is relatively small compared to the liver area and the boundary is usually blurred, the recognition difficulty is more challenging. In Figure 7, it can be seen that most of the cases have IOUs between 0.85 and 0.95, some are between 0.6 and 0.8, and a few are between 0.3 and 0.5, which can be considered as slightly poor in the direction of segmentation grasping.

**Figure 8:**
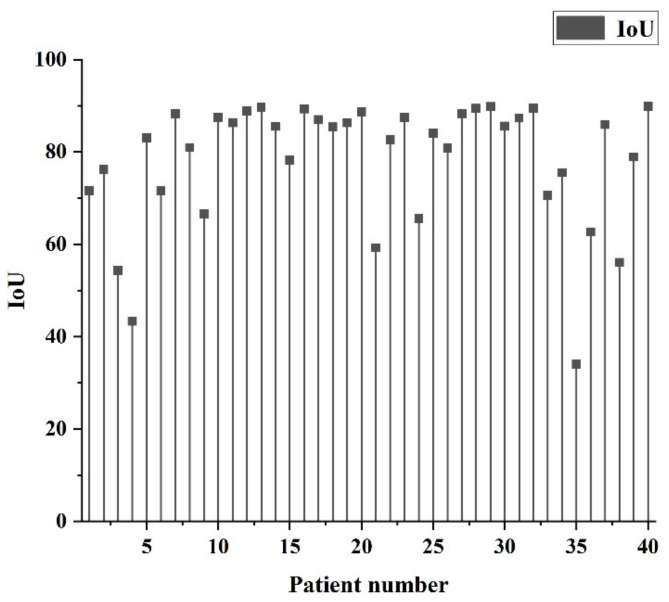
Distribution of IoU by patients after training with additional 30 hospital imaging data sets.

After mixing 30 additional hospital data into 101 LiTS data, a total of 131 data were trained for 50 epochs, and the values were obtained as shown in Table 3. The distribution of patient IoUs is shown in Figure 8. Comparing Table 3 with Table 2, it can be seen that there is a slight improvement in all indexes and a significant improvement especially in DSC, IoU, and AVGDIST of the lesion part. Further, from the AVGDIST, it can be seen that there is a better match for the segmented border part. Figure 8 shows that the number of cases with IoU below 0.5 after adding hospital data training has significantly decreased compared to Figure 7, which means that the original deviations in recognition have been identified and captured more correct liver blocks.

**Figure 9:**
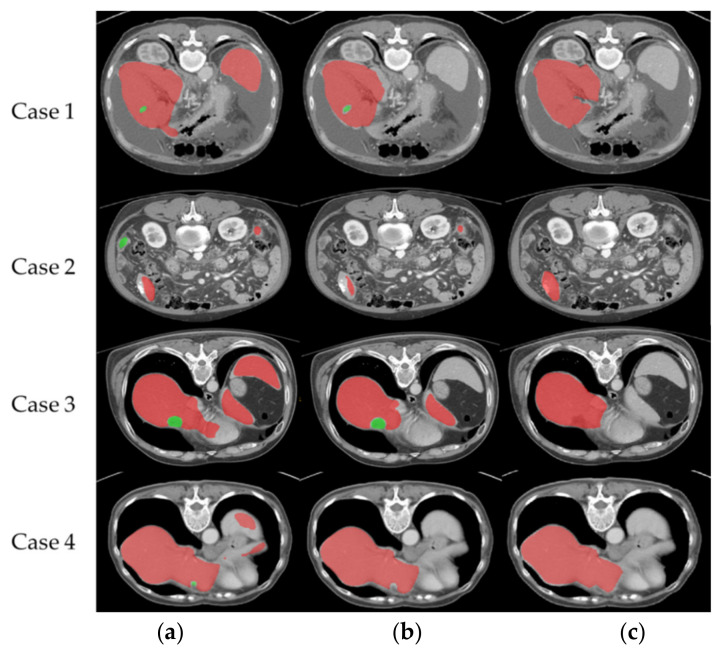
Comparative difference in segmentation of each case output: row (**a**) is the recognition output after training with the LiTS database, row (**b**) is the recognition output after training with 30 hospital data, and row (**c**) is the ground truth. (red is liver part, green is lesion part).

Figure 9 shows the comparative difference of segmentation of each case output. From case 1, it can be seen that the recognition output of the LITS database after training misidentified the parts of other surrounding organs as liver parts, which was corrected after importing the hospital data. As seen in case 2, the output of the LITS database incorrectly identified other organs in the non-liver block as lesions, which was improved after importing the hospital data. The same situation as in case 1 can be seen in case 3. As seen in case 4, in addition to the incorrect recognition output of the LITS database, which has captured more lesions below, other organs than the liver are also mistakenly recognized as liver. As a result, mixing with hospital imaging training can significantly reduce the results of liver and lesion misclassification.

### 4.4. Misidentification of Liver Volume and Pathological Data Comparison

Due to the pathological excision data of Kaohsiung Chang Gung Memorial Hospital, it was possible to compare the identified output values with the actual pathological excision records. The liver volume was calculated by superimposing it into the original medical image CT file using the identified segmentation and then importing it into ITK-SNAP to read the data from the original medical image. After combining the pathological data from the hospital, the liver volume was identified by H-denseUNet. It can be concluded that a 10.6% difference in the accuracy of liver volume can be achieved by importing hospital data into the training. Further, it is remarkable that the average error of identification and pathological anatomy data using the LiTS database was 9.6%, while the average error was reduced to 1% with the addition of hospital data.

**Figure 10:**
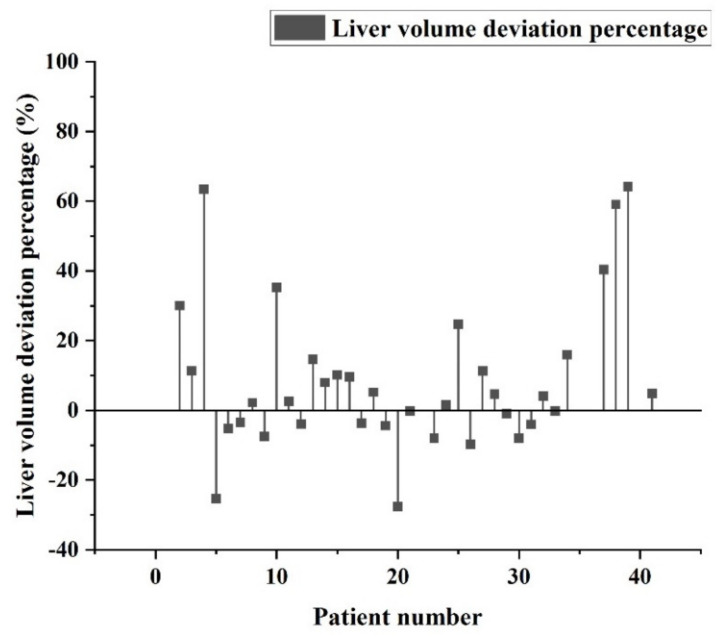
Percentage error between pathological data and identification data for individual patients.

**Figure 11:**
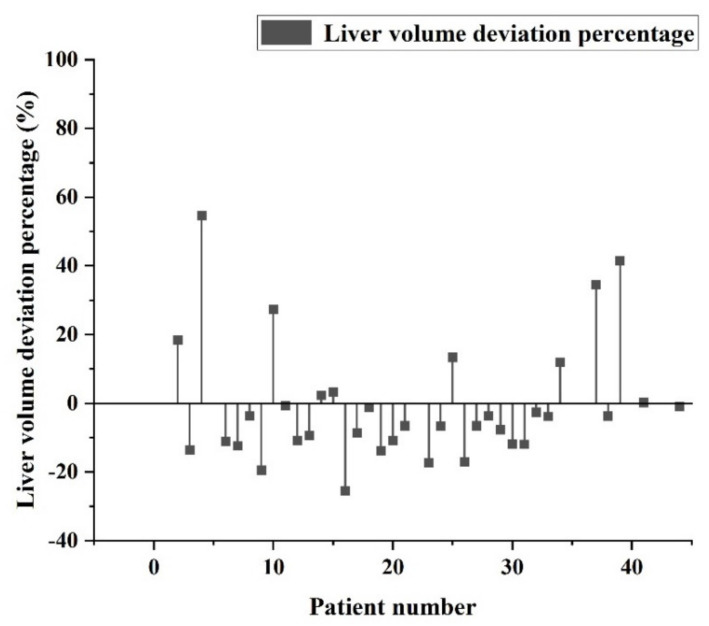
Percentage error between pathological data and identified data for individual patients after adding hospital data training.

The percentage error between pathological data and identification data for individual patients is shown in Figure 10. It can be seen that most of the error values are within −10% to +20%. After comparing the output data with the hospital pathology data, it can be seen that most of the errors are within the acceptable range. As a result, most of the data are still clinically referable, and only a few data have large deviation values (+40%). The percentage error between pathological data and identified data for individual patients after adding hospital data training is shown in Figure 11. It can be seen that most of the error values are within −15% to +5%. After mixing the hospital data into the training, it can be seen that the number of large deviations is reduced, and the overall deviation value is also reduced.

### 4.5. Misidentification of Tumor Length and Pathological Data Comparison

**Figure 12:**
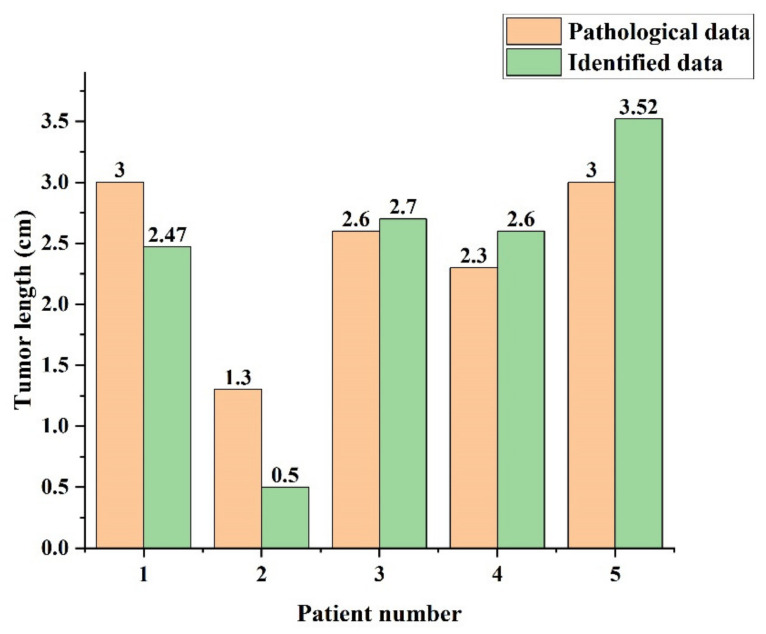
Comparison of tumor length by patients.

The independent pathological data and identification data of each patient are shown in Figure 12. The average error value of 0.43 cm and the standard deviation of the error value 0.502 were obtained by measuring the longest edge of each patient’s lesion using the LIFEx software and comparing it to the pathology data using the trained segmentation of the hospital data database. After tabulating the tumor lengths of the pathological data and the identified data, it can be seen that the tumor lengths of the patients have inconsistent errors between the pathological data and the identified data, but the identified data are still indicative. In particular, the percentage of error is lower when applied to larger tumor size.

## 5. Conclusions

According to the current trend of increasing number of studies on the application of deep learning to medical image recognition and increasing recognition rate, the application of deep learning results to medical centers or to patients for rapid detection and classification of conditions can be seen in the near future, and its recognition capability can surpass the visual observation of radiologists. However, few studies have been conducted to investigate the discrepancy between the application of the database and the error of pathological data. This study was conducted to understand the difference in the accuracy of the H-DenseUNet model in identifying liver and lesions that differed from the training database, and to show the improvement in recognition rate when hospital training data were added and a small amount of time was spent on training. A total of 70 patients were used in this study, of which 30 patients were used for training in the system and 40 patients were used as a testing group to test the recognition rate. After training, the AVGDIST in identifying the liver improved from 2.05 to 1.49, while the AVGDIST of the lesion improved from 7.92 to 6.25. The output segmentation was combined with the original CT image to create a 3D medical image with a combined fluoroscopic view of the lesion, allowing physicians to examine it more effectively. The average error of identification and pathological anatomy data using the LiTS database was 9.6%, while the average error was reduced to 1% with the addition of hospital data provided by Kaohsiung Chang Gung Hospital. Although the data in this study may be limited by the database and the experimental results may vary among different databases, it still provides a practical application of the error condition and the rare bias condition after comparing to the pathological data. In the future, it is hoped that the application can be applied to different hospital databases for recognition rate and pathology data discrepancies.

## Figures and Tables

**Table 1:** Lesion and liver volume values displayed by the ITK-Snap interface.

Label Name	Voxel Count	Volume (mm^3^)	Intensity Mean ± SD
0 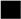 Clear Label	11386392	1.139 × 10^7^	−157.7376, ±96.9104
1 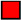 Label 1	409269	4.093 × 10^5^	110.5702, ±32.2686
2 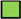 Label 2	819	819	49.2796, ±17.9682

**Table 2:** Accuracy of liver and lesion identification after training with the default LiTS database.

	ACC	DSC	IoU	AUC	AVGDIST
Liver	0.9897	0.853	0.764	0.967	2.05
Lesion	0.9872	0.525	0.408	0.781	7.92

**Table 3:** Accuracy of liver and lesion identification after training with an additional 30 hospital imaging data sets.

	ACC	DSC	IoU	AUC	AVGDIST
Liver	0.9904	0.871	0.783	0.951	1.49
Lesion	0.9907	0.611	0.488	0.81	6.25

## Data Availability

Restrictions apply to the availability of these data. Data was obtained from Kaohsiung Chang Gung Memorial Hospital and are available H.-Y.O., C.-C.L. and Y.-F.C. with the permission of Kaohsiung Chang Gung Memorial Hospital, Taiwan.
